# Supplementary material for: Saccharomyces boulardii (CNCM I-745) improves intestinal damage in sepsis by remodeling intestinal flora structure
Source: Front Cell Infect Microbiol. 2025 Jul 31;15:1602792. doi: 10.3389/fcimb.2025.1602792 (PMC12350329; doi:10.3389/fcimb.2025.1602792)
Supplement: Supplementary file 1 [file DataSheet1.zip › summary/summary/6_Taxonomy_community/1_Taxonomy_profiling/7_Species/Venn/venn_diagram_Group.All.pdf]

Flower Plot

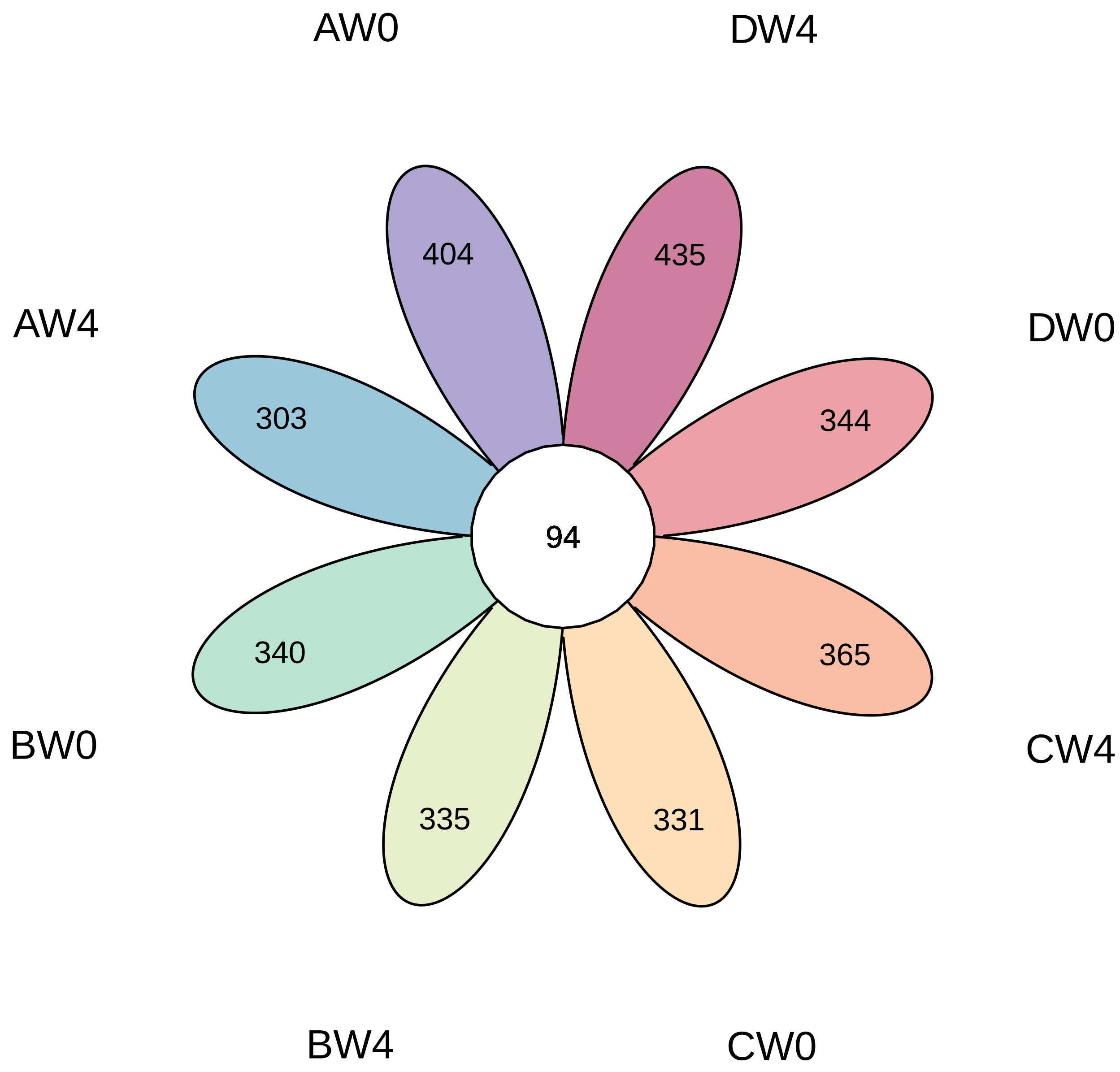

AW0
DW4
AW4
DW0
BW0
CW4
BW4
CW0
404
435
303
344
94
340
365
335
331
